# Caste‐ and pesticide‐specific effects of neonicotinoid pesticide exposure on gene expression in bumblebees

**DOI:** 10.1111/mec.15047

**Published:** 2019-03-06

**Authors:** Thomas J. Colgan, Isabel K. Fletcher, Andres N. Arce, Richard J. Gill, Ana Ramos Rodrigues, Eckart Stolle, Lars Chittka, Yannick Wurm

**Affiliations:** ^1^ School of Biological and Chemical Sciences Queen Mary University of London London UK; ^2^ School of Biological, Earth and Environmental Sciences University College Cork Cork Ireland; ^3^ Department of Life Sciences Imperial College London Ascot UK; ^4^ Present address: Institut für Biologie Martin‐Luther‐Universität Halle‐Wittenberg Halle Germany

**Keywords:** ecotoxicology, molecular diagnostics, neonicotinoid insecticides, nicotinic acetylcholine receptors, pollinator health, xenobiotics

## Abstract

Social bees are important insect pollinators of wildflowers and agricultural crops, making their reported declines a global concern. A major factor implicated in these declines is the widespread use of neonicotinoid pesticides. Indeed, recent research has demonstrated that exposure to low doses of these neurotoxic pesticides impairs bee behaviours important for colony function and survival. However, our understanding of the molecular‐genetic pathways that lead to such effects is limited, as is our knowledge of how effects may differ between colony members. To understand what genes and pathways are affected by exposure of bumblebee workers and queens to neonicotinoid pesticides, we implemented a transcriptome‐wide gene expression study. We chronically exposed *Bombus terrestris*colonies to either clothianidin or imidacloprid at field‐realistic concentrations while controlling for factors including colony social environment and worker age. We reveal that genes involved in important biological processes including mitochondrial function are differentially expressed in response to neonicotinoid exposure. Additionally, clothianidin exposure had stronger effects on gene expression amplitude and alternative splicing than imidacloprid. Finally, exposure affected workers more strongly than queens. Our work demonstrates how RNA‐Seq transcriptome profiling can provide detailed novel insight on the mechanisms mediating pesticide toxicity to a key insect pollinator.

## 1 INTRODUCTION

Social bees are important pollinators crucial for maintaining biodiversity and ecosystem stability (Garibaldi et al., 2013; Waser, Chittka, Price, Williams, & Ollerton, 1996). More than 85% of flowering plant species across the globe rely to some degree on animal pollination (Ollerton, Winfree, & Tarrant, 2011), and the agricultural industry values this pollination service at over €150 bn (Gallai, Salles, Settele, & Vaissière, 2009; Garibaldi et al., 2014; Klein et al., 2007). Reported insect pollinator declines are thus of worldwide concern (Aizen & Harder, 2009; Goulson, Nicholls, Botías, & Rotheray, 2015; Gill et al., 2016; Potts et al., 2016). Factors implicated as contributors to such declines include habitat loss, climate change, pathogens and in particular agricultural intensification (Brown & Paxton, 2009; Goulson et al., 2015; Vanbergen, 2013). Indeed, agricultural intensification has led to the increased usage of pesticides which can have unintended effects on social bees (Desneux, Decourtye, & Delpuech, 2007), with neonicotinoid pesticides having received particular attention (Gill, Ramos‐Rodriguez, & Raine, 2012; Goulson, 2013; Henry et al., 2012; Simon‐Delso et al., 2015; Whitehorn, O'Connor, Wackers, & Goulson, 2012).

Neonicotinoids are a popular class of neuroactive insecticides as they efficiently kill insect pests while having significantly lower toxicity to vertebrates (Jeschke, Nauen, Schindler, & Elbert, 2011; Matsuda et al., 2001). Furthermore, these insecticides are systemic: they are readily absorbed by plants and translocated to all tissues (Elbert, Haas, Springer, Thielert, & Nauen, 2008). A consequence of this, however, is that traces of neonicotinoids are detectable in the pollen and nectar of treated and contaminated flowering plants (David et al., 2016; Long & Krupke, 2016) that bees feed on (Rortais, Arnold, Halm, & Touffet‐Briens, 2005). Neonicotinoids target nicotinic acetylcholine receptors (nAChRs) which they bind to and thus excite; this can result in paralysis, convulsions and death (Matsuda et al., 2001). Controlled exposure experiments using honeybees and bumblebees have shown that exposure at comparable concentrations to those found in nectar and pollen can have sublethal effects on learning and memory (Siviter, Koricheva, Brown, & Leadbeater, 2018; Stanley, Smith, & Raine, 2015), cognition and problem solving (Baracchi, Marples, Jenkins, Leitch, & Chittka, 2017; Samuelson, Chen‐Wishart, Gill, & Leadbeater, 2016; Williamson & Wright, 2013), motor function (Drummond, Williamson, Fitchett, Wright, & Judge, 2016; Williamson, Willis, & Wright, 2014), foraging performance (Gill & Raine, 2014; Henry et al., 2012; Stanley, Russell, Morrison, Rogers, & Raine, 2016), navigation abilities (Fischer et al., 2014) and the immune system (Brandt, Gorenflo, Siede, Meixner, & Büchler, 2016; Brandt et al., 2017; Di Prisco et al., 2013). Despite the growing interest in the link between neonicotinoid exposure and toxicity to bees, we know little about the molecules and genes through which neonicotinoid action is mediated, or whether neonicotinoids may also affect “off‐target” processes that are not mediated by nAChRs.

An additional consideration is that neonicotinoids differ in manners that are only beginning to be characterized. Clothianidin and imidacloprid differentially affect distinct subpopulations of Kenyon cells cultured from bumblebee brains, suggesting that pathways by which they act differ (Moffat et al., 2016). In line with this, genome‐wide transcriptome profiling (RNA‐Seq) of honeybee brains showed differences between pesticides, with clothianidin exposure resulting in greater transcriptional changes than imidacloprid or thiamethoxam, including for metabolic and detoxification genes (Christen, Schirrmann, Frey, & Fent, 2018). Similarly to its use for diagnosing and classifying human diseases (Byron, Keuren‐Jensen, Engelthaler, Carpten, & Craig, 2016), RNA‐Seq can provide a holistic view of how pesticides affect genes underlying important processes, while also providing candidate genes for future functional studies.

Here, we aim to understand the impacts of neonicotinoid exposure on the bumblebee *Bombus terrestris,*a common wild Eurasian pollinator and the second‐most economically important bee pollinator species worldwide, using transcriptome profiling. Using a tightly controlled experimental design, we provided whole colonies with untreated food, or with food treated with one of two common neonicotinoids, clothianidin and imidacloprid. We performed RNA‐Seq gene expression profiling on heads of age‐controlled worker bumblebees in addition to colony queens, from colonies kept under controlled environmental conditions. The head is likely the key centre for mediation of the detrimental effects of neonicotinoids on behaviour and cognition because it contains important organs and tissues of the insect nervous system, in particular the brain, which contains an abundance of Kenyon cells, the neuronal cell type that neonicotinoids predominantly target within social bees (Moffat et al., 2016; Palmer et al., 2013). We exposed colonies for four days, a chronic exposure period after which neonicotinoid residues have previously been detected within the brains of exposed bumblebee workers (Moffat et al., 2015). We addressed the following questions: (a) Does neonicotinoid exposure lead to transcriptional changes in the head tissues of exposed bumblebees? (b) Do different neonicotinoids lead to different gene expression profiles? (c) Do workers and queens differ in their transcriptional response to neonicotinoids? Our work reveals pesticide‐ and caste‐specific effects on gene expression amplitude and splicing, providing detailed novel insight on the mechanisms mediating pesticide toxicity to bumblebees.

## 2 MATERIALS AND METHODS

### 2.1 Controlling colony size and worker age during colony rearing

We obtained 12 *Bombus terrestris audax* colonies containing a median of 56 workers (mean: 51.0; standard error (SE): 6.62, range: 15–93) from a commercial supplier (Agralan, UK). Each colony was randomly assigned to one of two identical controlled environment rooms maintained at 20°C and 60% humidity under constant red light illumination. Each colony was provided with ad libitum sucrose solution (40% w/w prepared using distilled water) and honeybee‐collected pollen (Agralan, UK) three times per week (Monday 2 g, Wednesday 2 g and Friday 3 g). It is relevant to note that this pollen lacks an organic certification; thus, it may contain trace amounts of xenobiotics, such as neonicotinoids or other insecticides. Therefore, we consider our experimental colonies to have been exposed to higher doses of the two pesticides in comparison with our control colonies.

Six days (144 hr) before starting the experimental treatment, we removed and tagged up to four newly eclosed workers per colony with a numbered Opalith tag (Abelo, UK). Once tagged, we placed them back into the colony. We also standardized the size of each colony by removing workers so that each colony contained the colony queen and a median of 20 workers (mean: 19.7; SE: 0.41; range: 15–21). For this, we marked each untagged worker in the colony with a white, nontoxic pen (Uniball Uni Posca). This enabled subsequent differentiation between old workers and newly eclosed workers. To maintain the number of workers in the colony constant, we removed marked (i.e. older) workers when unmarked (i.e.younger) workers eclosed, and immediately marked the new workers with the white pen.

### 2.2 Preparation of sucrose solutions containing neonicotinoid pesticides

We prepared stock solutions of each pesticide by dissolving either analytical grade clothianidin or imidacloprid (Sigma Aldrich, UK) in acetone to a concentration of 1.0 × 10^−3^ g/ml. We serially diluted the stock solution using 40% sucrose solution to produce a 1.0 × 10^−6^ g/ml working solution, which was stored in the dark at 4°C for a maximum of 4 days. The working solution was then further diluted with 40% sucrose solution to produce a final concentration of 7.5 × 10^−9^ g/ml. We prepared solutions no more than 1 hr before providing them to the bumblebee colonies. As the mass of 1 L of 40% sucrose is 1,160 g and contained 7.5 × 10^–6^ g of pesticide, each sucrose solution contained 6.47 parts per billion (ppb) of pesticide, which is within the range that bees are considered to be exposed to within the field (Supporting information Table S1).

### 2.3 Exposure of colonies to neonicotinoid‐laced sucrose

We randomly assigned each colony to one of the three treatment groups: control (*n* = 4), clothianidin (*n* = 4) or imidacloprid (*n* = 4). For the purpose of measuring changes in worker gene expression in response to neonicotinoid exposure, we only used workers age‐controlled to 10 days post‐eclosion (Supporting information Figure S1). At the start of day six, we removed the initial sucrose feeders and any remaining pollen. We provided each colony with its allocated treatment and 2 g pollen; we replaced the food of each colony after 24 and 48 hr; and we ended the experiment after 96 hr of exposure. At the end of the experiment, we transferred the 10 day‐old Opalith tagged workers and the colony queen into individual 2 and 5 ml Eppendorf tubes, respectively, snap froze them in liquid nitrogen and then stored the tubes at −80°C.

### 2.4 RNA extractions, library preparations and high throughput sequencing

We extracted RNA from the colony queen and from one worker per colony from 12 colonies (*n* = 24 individuals). For this, we removed bumblebee‐containing cryotubes from −80°C storage and kept them on dry ice. Using sterilized forceps, we transferred each bumblebee from the housing cryotube onto a sterilized 5 ml Petri dish that had been chilled on ice. Using a new sterile blade for each sample, we removed the head and transferred it into a new 2 ml homogenization tube containing 150 µl of Tri reagent (Sigma, UK). The contents of each tube were then frozen on dry ice and returned to −80°C storage. For total RNA extraction, each individual sample was removed from storage and kept on ice. To each tube, we added 0.2 g zirconium silicate (ZS) beads (Sigmund Lindner GmbH, Germany) and homogenized each sample using a FastPrep‐96 high throughput homogenizer using two cycles of 45 s at 281.7 x g. After homogenization, each sample was visually examined to ensure thorough sample disruption. We added 850 µl of Tri reagent to each tube and incubated at room temperature for 5 min to allow for complete dissociation of nucleoprotein complexes. We isolated total RNA using chloroform following the manufacturer's recommendations. We precipitated total RNA using isopropanol and performed a wash using molecular‐grade ethanol. To remove potential phenol and ethanol contamination, we further purified the extracted RNA for each individual using the RNeasy MiniPrep kit (Qiagen, UK). Finally, we removed residual DNA using RNase‐free DNase I (Qiagen, UK). We quantified total RNA using a Qubit RNA Broad‐Range (BR) Assay kit (Invitrogen, UK).

We prepared sequencing libraries (*n* = 24) using the Illumina TruSeq stranded mRNA library preparation kit. For each library, we used a starting concentration of 1.5 µg of total RNA. We purified each library using AMPure XL beads (Beckman Coulter, UK) and quantified library size using a TapeStation 2200 (Agilent, UK). Using equal concentrations of each library, we created a single pooled library. We sequenced the pooled library on Illumina NextSeq 500 and HiSeq 4000 generating ~ 129.72 million reads of 76 bp and ~314.6 million reads of 50 bp. We thus obtained a mean of 18.51 million reads per sample (min: 9.84 million; max: 23.89 million reads per sample) (Supporting information Table S2).

### 2.5 Quality assessment of Illumina RNA‐Seq reads

We assessed the quality of raw reads using two primary measures. First, we initially assessed sequence quality using fastqc (version 0.11.3; Andrews, 2010) to identify potential adapter contamination and low base qualities. Subsequently, we aligned raw reads against the *Bombus terrestris* reference genome assembly (GCF_000214255.1; Sadd et al., 2015) using HISAT2 (version 2.1.0; Kim, Langmead, & Salzberg, 2015). We calculated mapping statistics for the resulting alignment files using qualimap (version 2.2.1; García‐Alcalde et al., 2012) and visualized the output summaries using multiqc (version 0.7; Ewels, Magnusson, Lundin, & Käller, 2016). A summary of raw sequence quality and alignment statistics is provided in Supporting information Appendix [Link], [Link]. For each sample, >88% of reads mapped uniquely to the *B. terrestris* genome; all RNA‐Seq libraries were of high quality and retained for analysis.

### 2.6 Identifying pesticide exposure effects on gene expression amplitude

We quantified transcript abundance for each sample by pseudoaligning reads (kallisto, verion 0.44.1; Bray, Pimentel, Melsted, & Pachter, 2016; run parameters: ‐‐single ‐l 300 ‐s 20) to predicted transcripts from the *B. terrestris* genome (Ensembl release version 40). To facilitate reanalysis of these data, we provide raw estimated counts for all samples in Supporting information Table S3. Estimated counts were summarized per gene using tximport (version 1.6.0; with countsFromAbundance = "no"; Soneson et al., 2015) and imported into DESeq2 (version 1.14.1; Love, Huber, & Anders, 2014). We created a DESeq2 object containing the entire data set. We used DESeq2 Wald tests to identify genes that were differentially expressed between each pesticide treatment and the control colonies (Benjamini–Hochberg (BH) adjusted *p*‐value < 0.05). As an additional measure of confidence, we identified >80% of these statistically significant genes with DESeq2 when using gene‐level counts generated by the HISAT2‐HTSeq pipeline as input. Similar to the kallisto‐based analysis, the HISAT2‐based approach identified caste‐ and pesticide‐specific changes in bumblebee gene expression. Using this approach, we identified greater amplitude changes in gene expression in workers in comparison with queens. In addition, for both castes, clothianidin exposure resulted in greater gene expression changes than imidacloprid. Additional information on the specific findings of this comparative analysis is provided in the Supplemental Information.

### 2.7 Identifying pesticide exposure effects on alternative splicing

We aligned raw reads against the *B. terrestris*genome (Ensembl release version 40) using the splice aware aligner HISAT2 (version 2.1.0; Kim et al., 2015) and obtained read counts for each exon using HTSeq (version 0.9.1; with ‐‐stranded = “reverse”; Anders, Pyl, & Huber, 2015). To facilitate reanalysis of these data, we provide exon‐level counts in Supporting information Table S4. We created DEXSeq objects and analysed differential exon usage for each pesticide treatment in comparison with control individuals using DEXSeq (version 1.20.2; Reyes et al., 2013) informed by the Ensembl GTF file.

### 2.8 Gene Ontology enrichment analysis

For each gene, we identified the *Drosophila melanogaster*ortholog from Ensembl Metazoa Biomart (Kinsella et al., 2011) and used its Gene Ontology (GO) annotations because little functional information exists for *B. terrestris*. To test whether any Gene Ontology terms were overrepresented among the most highly differentially expressed genes in response to pesticide exposure, we sorted all *B. terrestris*genes by raw *p‐*value (because of edge effects associated with adjusted *p*‐values) and performed a rank‐based test for each GO term. For this, we used Kolmogorov–Smirnov tests in topGO (version 2.34.0; with the "weight01" algorithm and nodeSize = 100; Alexa & Rahnenfuhrer, 2018).

## 3 RESULTS

### 3.1 Clothianidin exposure leads to differential gene expression in worker and in queen bumblebees

**Figure 1:**
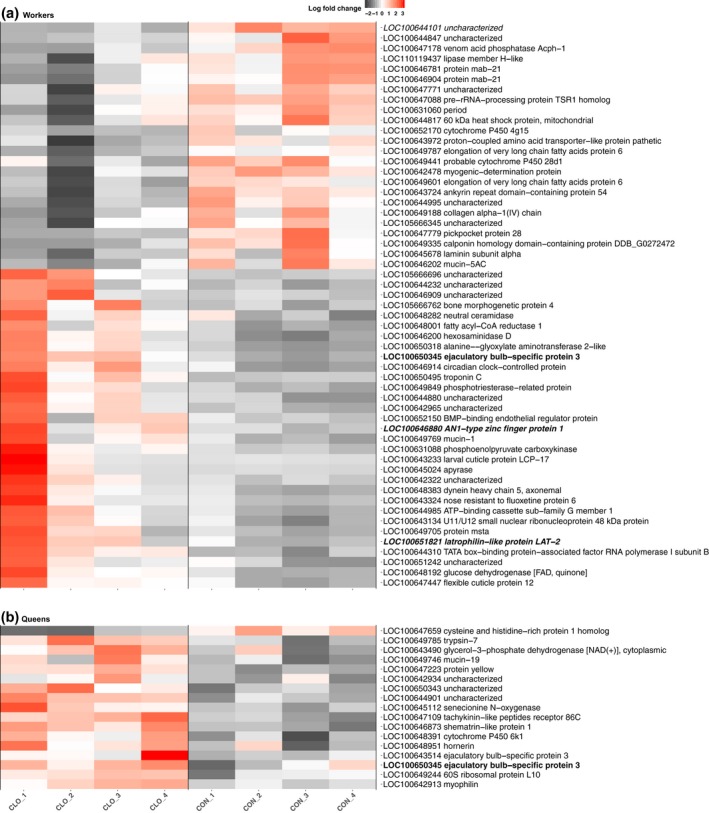
Chronic clothianidin exposure leads to gene expression changes in bumblebee workers and queens. Heatmaps displaying genes differentially expressed in workers (a; *n* = 55) and in queens (b; *n* = 17) between clothianidin‐exposed and control colonies. For each differentially expressed gene, we show the log fold change for each biological replicate, as well as the gene identifier and NCBI's functional gene description. The single gene differentially expressed in both castes is indicated in bold. The single gene also differentially expressed in imidacloprid‐exposed workers is indicated in italics. The two genes identified to be differentially expressed and alternatively spliced within clothianidin‐exposed workers are indicated in bold and italics [Colour figure can be viewed at wileyonlinelibrary.com]

We determined that 55 genes are significantly differentially expressed in workers in response to clothianidin exposure compared to workers fed on the control diet (BH adjusted *p*‐value < 0.05, Figure 1(a); Supporting information Table S5). Among these genes, 31 (62%) were more highly expressed after exposure; this pattern was nonsignificant (binomial test *p*‐value = 0.4). Several of the differentially expressed genes are involved in key biological processes, and orthologs to some of the genes have been shown to be differentially expressed in other species exposed to pesticides (see Discussion). In particular, three of the 55 genes identified were among the 244 genes differentially expressed in the brains of honeybee workers exposed to clothianidin (Christen et al., 2018), suggesting that certain similar biological processes may be affected across species. Two of these genes, *mab‐21* (LOC100646781), a putative developmental gene, and *proton‐coupled amino acid transporter‐like protein pathetic* (LOC100643972), a putative solute transporter gene, had reduced expression in response to exposure in both experiments. Intriguingly; however, *glucose dehydrogenase* (LOC100648192) was more highly expressed in response to clothianidin in our bumblebees but had reduced expression after exposure in honeybees (Christen et al., 2018), also indicating that a single pesticide can have opposing effects on different species.

We also investigated whether clothianidin exposure affected expression profiles of colony queens. Seventeen genes were significantly differentially expressed (BH adjusted *p*‐value < 0.05, Figure 1(b); Supporting information Table S5), and unlike in workers, we found a strong pattern of increased expression: only one of these genes had lower expression after exposure (binomial test *p*‐value < 10^−3^). Among the more highly expressed genes in the clothianidin‐exposed queens were genes coding for a putative neurohormone receptor, *tachykinin‐like peptides receptor 86C* (LOC100647109), a developmental gene, *protein yellow* (LOC100647223) and two putative odorant binding proteins (LOC100643514; LOC100650345).

Strikingly, there was almost no overlap between the lists of genes differentially expressed in the two castes, suggesting that the phenotypic effects and susceptibility to exposure differ between castes. The one gene that was differentially expressed in both castes in response to clothianidin is LOC100650345, which contains an odorant binding protein A10 domain (IPR005055), suggesting it may play a role in the transport or perception of semiochemicals.

### 3.2 Clothianidin exposure leads to alternative splicing in worker and in queen bumblebees

Clothianidin exposure resulted in the significant alternative splicing of 45 genes in exposed workers (BH adjusted *p*‐value < 0.05, Supporting information Figure S2). Two genes (LOC100646880; LOC100651821) were both differentially expressed and alternatively spliced after clothianidin exposure (Figure 1(a)). By comparison, in queens, we identified no evidence of alternative splicing in response to clothianidin exposure.

### 3.3 Imidacloprid exposure induced weaker transcriptional changes than clothianidin

We also investigated changes in gene expression in response to imidacloprid exposure. Intriguingly, we found no differences in gene expression amplitude between exposed and control queens, but eight genes were alternatively spliced (Table 1). Thus in queens, imidacloprid exposure affects half as many genes as clothianidin and through a different mechanism.

**Table 1:** Genes with differential expression after neonicotinoid exposure in bumblebees. For each treatment, the number of genes with differential amplitude and differential splicing per caste is shown

Treatment	Caste	Genes with differential amplitude	Genes with differential splicing
Clothianidin	Workers	55	45
	Queens	17	0
Imidacloprid	Workers	1	1
	Queens	0	8

In workers, only one gene was differentially expressed (LOC100644101), and a single different gene was alternatively spliced in response to imidacloprid exposure (LOC100649110). Interestingly, these two genes had similar expression patterns in terms of differential expression and alternative splicing, respectively, in clothianidin‐exposed workers, highlighting a potential generic molecular response to neonicotinoid exposure in *B. terrestris* workers. LOC100644101 codes for a protein with homologs throughout the Hymenoptera but no known functional domains. In comparison, LOC100649110 codes for a protein with a predicted cytochrome P450, E‐class, group 1 functional domain (IPR002401), suggestive of a role in the metabolism of exogenous substances or endogenous physiologically‐active compounds.

## 4 DISCUSSION

Understanding the sublethal effects of pesticide exposure on beneficial organisms such as insect pollinators is important in order to assess the risks posed by pesticides. Focusing on the molecular‐genetic level, we carried out genome‐wide mRNA‐sequencing of the heads of bumblebees chronically exposed for four days to one of two widely used neonicotinoid insecticides, clothianidin and imidacloprid. We reveal three major novel trends: (a) head tissues of bumblebee workers and queens exhibit significant changes in gene expression amplitude and alternative splicing due to clothianidin or imidacloprid exposure; (b) clothianidin had stronger effects than imidacloprid on gene expression; (c) the worker and queen castes intriguingly differed in their response to neonicotinoid exposure, with both neonicotinoids leading to greater transcriptional changes in workers than in queens. Our results provide high‐resolution insight into the molecular‐genetic pathways by which neonicotinoids affect colony members. Some of these effects likely occur downstream of the nACh receptors that neonicotinoids target. However, some of the effects we see could be due to interactions between the pesticide and “off‐target” receptors or other cellular components within the head or other body parts of the exposed bumblebees.

We are wary of providing detailed potential interpretations regarding individual genes or pathways seen in a single study because most of what we know about bumblebee genes is bioinformatically inferred rather than being demonstrated experimentally. However, clothianidin and imidacloprid have been observed to cause mitochondrial depolarization in Kenyon cells of social bee brains (Moffat et al., 2016, 2015). Differentially expressed genes associated with mitochondrial function such as *alanine‐glyoxylate aminotransferase* and *phosphoenolpyruvate carboxykinase* are thus strong candidate genes mediating such effects. The second of these genes also has increased expression in imidacloprid‐exposed honeybee larvae (Derecka et al., 2013) and in dichlorodiphenyltrichloroethane (DDT)‐exposed *Drosophila melanogaster* (King‐Jones, Horner, Lam, & Thummel, 2006), suggesting a general mechanism of response to toxins across taxa. Due to the role of *phosphoenolpyruvate carboxykinase* in glycolysis and gluconeogenesis pathways, differential expression of this gene has been suggested to be associated with changes in energy use in response to a xenobiotic challenge (King‐Jones et al., 2006), as well as starvation (Zinke, Kirchner, Chao, Tetzlaff, & Pankratz, 1999). Gene Ontology terms associated with carbohydrate and lipid metabolism were also enriched in clothianidin‐exposed workers and queens (Supporting information Figure S3; Table S6), suggesting potential changes in energy usage.

Clothianidin and imidacloprid belong to the chemical group of N‐nitroguanidines (Jeschke & Nauen, 2008), and within species, the two pesticides are generally thought to have similar toxicities based on toxicity values such as LD_50_in honeybee (Brandt et al., 2016; Iwasa, Motoyama, Ambrose, & Roe, 2004) and the western chinch bug (Stamm et al., 2011). However, some studies report higher lethality of clothianidin than imidacloprid in the honeybee *Apis mellifera* (Laurino, Manino, Patetta, & Porporato, 2013), the bumblebee *B. impatiens*, the alfalfa leafcutter bee *Megachile rotundata* and in the orchard mason bee *Osmia lignaria* (Scott‐Dupree, Conroy, & Harris, 2009). Furthermore, clothianidin has been shown to depolarize bumblebee neural mitochondria more rapidly than imidacloprid (Moffat et al., 2015). Our study found that chronic clothianidin exposure affected gene expression much more strongly than imidacloprid. This further mirrors findings on the honeybee brains (Christen et al., 2018) and overall suggests that clothianidin indeed has stronger transcriptional effects than imidacloprid. There may be technical and biological reasons for why we found relatively few effects of imidacloprid. We used a low concentration (6.47 ppb) of both pesticides, considered to be within the range foraging bees are exposed to in the field (Supporting information Table S1), rather than the high doses often used to demonstrate strong effects. It is also possible that the sample size we used lacked the power to detect subtle, but potentially important effects, of imidacloprid on gene expression. Furthermore, we examined gene expression at a single time point after four days of chronic exposure yet the chronic effects of exposure may differ between pesticides. Indeed, at an extreme level, the phenylpyrazole insecticide fipronil accumulates within honeybees, leading to strong effects over time (Holder, Jones, Tyler, & Cresswell, 2018). The study of the effects of long‐term exposure of different pesticide classes on bumblebees, as well as associated gene expression, is required. Finally, it is plausible that the different neonicotinoids have disproportionate effects on gene expression on different sets or subsets of neurons. Detecting such particularly localized effects can be challenging because we obtained for each gene the average expression across all of the cells in the entire head.

A key trait of social bees such as honeybees and most bumblebees is that colonies include a queen and workers that differ in morphology and physiology and have complementary behaviours essential for colony fitness. Thus, castes may differ in how they are affected by pesticide exposure. Our genome‐wide transcriptome RNA‐Seq profiling approach found that only one gene, LOC100650345, was differentially expressed in both workers and queens. We know little about this gene other than it has been observed to be expressed in queen haemolymph (Sadd et al., 2015), and carries an odorant binding domain, suggesting that it may play a role in the transportation of semiochemicals such as odours and pheromones in the haemolymph. Its role may be conserved as a homologous gene in the whitefly *Bemisia tabaci* is also upregulated after exposure to the neonicotinoid thiamethoxam (Liu et al., 2016, 2014). Several general functions (Gene Ontology terms) were shared by workers and queens, including oxidation–reduction process, glucose metabolic process and single‐organism catabolic process (Supporting information Figure S3). However, there were also marked differences. In queens, differentially expressed genes included functions related to the determination of lifespan, lipid metabolic process and ion transport, while genes affected in workers included genes involved in regulation of developmental growth, neuron projection guidance and regulation of the Notch signalling pathway (Supporting information Figure S3). A previous study reported that expression of cytochrome P450 genes, a family of genes typically involved in chemical detoxification, is affected by imidacloprid in honeybees (Chaimanee, Evans, Chen, Jackson, & Pettis, 2016). In line with this, two cytochrome P450 CYP9Q subfamily genes in bumblebees metabolize the neonicotinoid thiacloprid but not imidacloprid (Manjon et al., 2018). Intriguingly, we found no effect of neonicotinoid exposure on either of these genes, suggesting that the genes do not code for products that metabolize clothianidin, or that they function on different timescales or tissues than our study focused on. However, three other putative cytochrome P450 genes, LOC100652170, LOC100649441 and LOC100648391, respectively, members of the CYP4, CYP6 and CYP9 subfamilies, were differentially expressed after clothianidin exposure (Figure 1), while one CYP6 family member (LOC100649110) was alternatively spliced in workers in response to both neonicotinoids, thus providing additional candidates for future work investigating the defence of bees against neonicotinoid pesticides. Members of these families have higher expression within the hypopharyngeal and mandibular glands of honeybee foragers in comparison with nurses suggestive of a role in the metabolism of xenobiotic and phytochemicals that foragers are exposed to during natural foraging trips (Vannette, Mohamed, & Johnson, 2015).

Multiple biological and technical reasons could explain differences between castes. First, workers forage for food, care for brood, and build, maintain and defend the nest while the queen lays batches of eggs daily. Additionally, queens live up to a year while *B. terrestris* workers live two months on average (Alford, 1975). Their behaviours and physiologies thus fundamentally differ, and selection will over time have shaped response thresholds to external challenges in caste‐specific manners. Second, it is plausible that exposure differed between castes. Our study was designed to prevent artefactual expression differences due to variation in colony size or the absence of the queen: we maintained entire colonies. This did compromise, however, being able to precisely control neonicotinoid dosage. Potential variation in exposure could come from differences in feeding behaviours between and within castes, such as feeding directly from the feeder or from nectar pots. Further sources of biological noise can come from intercolony variation. For example, colonies have baseline inherited differences in which alleles they carry, in gene expression levels, in response thresholds for behaviours such as feeding rates, in susceptibility to introduced compounds, and other biological differences. The effects of some such differences are likely responsible for the variation in gene expression among the four control colonies (Figure 1). To account for such variation, future studies of ecologically relevant organisms will benefit from strong replication at the appropriate (e.g. colony) level. A final source of biological noise comes from ages: we precisely controlled the ages of bumblebee workers to 10 days post‐eclosion but were unable to determine the ages of queens because commercially supplied colonies come with no such information. An alternate explanation for finding differences between queens and workers may be technical, and our study focuses on gene expression in heads. Indeed, heads include multiple tissues that differ in relative size between queens and workers (e.g. queens possess fully developed corpora allata while workers do not (Röseler & Röseler, 1978)). Such allometric differences could affect our estimation of relative impacts on gene expression (Johnson, Atallah, & Plachetzki, 2013). The use of alternative tissues, such as the digestive tract or malpighian tubules, key organs in xenobiotic metabolism, may provide additional insights into how castes respond to neonicotinoid exposure. Therefore, future expression studies will benefit from approaches targeting multiple specific tissues or cell types.

The majority of studies on the molecular effects of insecticides have focused on the expression of their direct target sites, such as ligand‐ and voltage‐gated ion channels, or on a priori candidate metabolic enzymes involved in detoxification of xenobiotic compounds. Whole transcriptome profiling studies such as ours have highlighted additional genes with altered expression in response to pesticide exposure. Some of the genes affected by clothianidin exposure in our study have also been affected by neonicotinoids or other pesticides in other studies and species. These include muscular genes such as *troponin* and *calponin* (Kimura‐Kuroda et al., 2016; Lewis, Szilagyi, Gehman, Dennis, & Jackson, 2009; Wang et al., 2015) and metabolic enzymes such as *glucose dehydrogenase* (Christen et al., 2018) and *hexosaminidase D* (Qi et al., 2018; Yang, Liu, Liu, Qu, & Qian, 2008). At a different level, cellular transport genes such as the ABC transport family (Dermauw & Van Leeuwen, 2014), one member of which was differentially expressed in our study, have been suggested to provide tolerance of neonicotinoids, such as imidacloprid, acetamiprid and thiacloprid, with greater mortality identified for neonicotinoid‐exposed honeybee larvae treated with an ABC inhibitor (Hawthorne & Dively, 2011). Furthermore, G protein‐coupled receptors, such as *tachykinin‐like peptides receptor 86C*, which has increased expression in clothianidin‐exposed queens in our study, have been identified as potential targets for the development of novel pesticides (Audsley & Down, 2015). Further work will indicate to which extent the genes and pathways we have identified represent useful biomarkers of pesticide toxicity. Finally, we suggest that some of the other changes we identified in the expression of specific genes or pathways, such as genes under circadian control, may mediate phenotypic effects of pesticide exposure that remain to be fully characterized.

## 5 CONCLUSIONS

Our study represents an important step towards understanding the diversity of effects of chronic exposure to clothianidin and imidacloprid. In addition to identifying caste‐ and pesticide‐specific effects, we provide lists of candidate genes for future research to improve our understanding of the impact of pesticides on bumblebee health. Our understanding of the significance of these genes and others will benefit from increased tissue profiling to identify tissue‐specific responses, investigation of the effects of other pesticide compounds and understanding of how effects of exposure change over time. Such detailed understanding can ultimately be helpful in classifying and quantifying the relative effects of pesticides on target pest species and beneficial species. Much like RNA‐Seq has changed the way we diagnose and understand human disease (Byron et al., 2016), we thus expect it to become a valuable tool during the development as well as regulatory evaluation of novel pesticides.

## AUTHOR CONTRIBUTIONS

This study is part of the postdoctoral research of T.J.C. under the supervision of Y.W. R.J.G., L.C. and Y.W. conceived the idea; T.J.C., A.N.A., R.J.G. and Y.W. designed the experiment; A.N.A. and A.R.R. performed the exposure experiments; T.J.C. and E.S. generated the RNA‐Seq libraries; T.J.C., I.K.F. and Y.W. analysed the data; T.J.C., I.K.F., A.N.A., R.J.G. and Y.W. wrote the manuscript. All authors edited the manuscript and gave final approval for publication.

## Data Availability

Raw sequence data files are deposited in the NCBI short read archive (Accession ID: PRJNA508397). Scripts underpinning the analysis of differential expression, differential exon usage and Gene Ontology term enrichment are archived on Github (https://github.com/wurmlab/Bter_neonicotinoid_exposure_experiment). Raw sequence counts for each sample are provided in the Supplemental Information.
