# Supplementary material for: Caste‐ and pesticide‐specific effects of neonicotinoid pesticide exposure on gene expression in bumblebees
Source: Mol Ecol. 2019 Mar 6;28(8):1964–74. doi: 10.1111/mec.15047 (PMC6563198; doi:10.1111/mec.15047)
Supplement: Supplementary file 7 [file MEC-28-1964-s007.html]

MultiQC Report


# Toggle navigation v0.7

- QualiMap
  - Coverage Histogram
  - Genome Fraction Coverage
  - GC-content distribution
- FastQC
  - Sequence Quality Histograms
  - Per Sequence Quality Scores
  - Per Base Sequence Content
  - Per Sequence GC Content
  - Per Base N Content
  - Sequence Length Distribution
  - Sequence Duplication Levels
  - Adapter Content

Toolbox

### MultiQC Toolbox

#### Highlight Samples

+

Regex mode off
help
 Clear

#### Rename Samples

+

Click here for bulk input.

Paste two columns of a tab-delimited table here (eg. from Excel).

First column should be the old name, second column the new name.

Add

Regex mode off
help
 Clear

#### Show / Hide Samples

Hide matching samples

Show only matching samples

+

Regex mode off
help
 Clear

#### Export Plots

- Images
- Data

px

px

Aspect ratio

PNG
JPEG
SVG

Plot scaling

X

Download the raw data used to create the plots in this report below:

Format:

Tab-separated
Comma-separated
JSON

Note that additional data was saved in `multiqc_data` when this report was generated.

---

##### Choose Plots

 All
 None

---

   Download Plots

If you use plots from MultiQC in a publication or presentation, please cite:

> **MultiQC: Summarize analysis results for multiple tools and samples in a single report**  
> *Philip Ewels, Måns Magnusson, Sverker Lundin and Max Käller*  
> Bioinformatics (2016)  
> doi: 10.1093/bioinformatics/btw354  
> PMID: 27312411

#### Save Settings

You can save the toolbox settings for this report to the browser.

 Save


---

#### Load Settings

Choose a saved report profile from the dropdown box below:

[ select ]

Load
 Delete

#### About MultiQC

This report was generated using MultiQC, version 0.7

You can see a YouTube video describing how to use MultiQC reports here:
https://youtu.be/qPbIlO\_KWN0

For more information about MultiQC, including other videos and
extensive documentation, please visit http://multiqc.info

You can report bugs, suggest improvements and find the source code for MultiQC on GitHub:
https://github.com/ewels/MultiQC

MultiQC is published in Bioinformatics:

> **MultiQC: Summarize analysis results for multiple tools and samples in a single report**  
> *Philip Ewels, Måns Magnusson, Sverker Lundin and Max Käller*  
> Bioinformatics (2016)  
> doi: 10.1093/bioinformatics/btw354  
> PMID: 27312411

# 

A modular tool to aggregate results from bioinformatics
analyses across many samples into a single report.

Report generated on 2018-12-22, 00:12 based on data in:
`/data/scratch/btw928/2018-12-20_hisat2_fastqc/all_qc_files`

---

×
don't show again

**Welcome!** Not sure where to start?  
Watch a tutorial video
  *(6:06)*

## General Statistics

 Copy table

 Configure Columns

 Sort by highlight
Showing 144/144 rows and 7/15 columns.

| Sample Name | Avg. GC | ≥ 50X | ≥ 30X | ≥ 10X | ≥ 05X | ≥ 01X | Coverage | % Aligned | Aligned | Total Reads | % Dups | % GC | Length | % Failed | M Seqs |
| --- | --- | --- | --- | --- | --- | --- | --- | --- | --- | --- | --- | --- | --- | --- | --- |
| 2016-Bter-CLO-C02-4-Q-head.D702\_D503.54243303.s\_fc.R1 |  |  |  |  |  |  |  |  |  |  | 57.9% | 41% | 76 | 25% | 5.6 |
| 2016-Bter-CLO-C02-4-Q-head.D702\_D503.HM3WHBBXX.s\_6.R1 |  |  |  |  |  |  |  |  |  |  | 66.5% | 42% | 50 | 33% | 2.1 |
| 2016-Bter-CLO-C02-4-Q-head.D702\_D503.HM3Y5BBXX.s\_6.R1 |  |  |  |  |  |  |  |  |  |  | 67.5% | 42% | 50 | 25% | 4.6 |
| 2016-Bter-CLO-C02-4-Q-head.D702\_D503.HMWT2BBXX.s\_3.R1 |  |  |  |  |  |  |  |  |  |  | 70.5% | 42% | 50 | 33% | 4.7 |
| 2016-Bter-CLO-C02-4-W1-head.D702\_D505.54242293.s\_fc.R1 |  |  |  |  |  |  |  |  |  |  | 54.1% | 40% | 76 | 25% | 7.2 |
| 2016-Bter-CLO-C02-4-W1-head.D702\_D505.HM3WHBBXX.s\_6.R1 |  |  |  |  |  |  |  |  |  |  | 58.0% | 42% | 50 | 33% | 1.6 |
| 2016-Bter-CLO-C02-4-W1-head.D702\_D505.HM3Y5BBXX.s\_6.R1 |  |  |  |  |  |  |  |  |  |  | 61.6% | 42% | 50 | 25% | 5.2 |
| 2016-Bter-CLO-C02-4-W1-head.D702\_D505.HMWT2BBXX.s\_3.R1 |  |  |  |  |  |  |  |  |  |  | 64.6% | 42% | 50 | 33% | 5.4 |
| 2016-Bter-CLO-C27-4-Q-head.D703\_D501.54247262.s\_fc.R1 |  |  |  |  |  |  |  |  |  |  | 51.0% | 40% | 76 | 25% | 2.5 |
| 2016-Bter-CLO-C27-4-Q-head.D703\_D501.HM3WHBBXX.s\_6.R1 |  |  |  |  |  |  |  |  |  |  | 69.7% | 41% | 50 | 33% | 2.8 |
| 2016-Bter-CLO-C27-4-Q-head.D703\_D501.HM3Y5BBXX.s\_6.R1 |  |  |  |  |  |  |  |  |  |  | 69.8% | 41% | 50 | 25% | 6.4 |
| 2016-Bter-CLO-C27-4-Q-head.D703\_D501.HMWT2BBXX.s\_3.R1 |  |  |  |  |  |  |  |  |  |  | 72.8% | 41% | 50 | 33% | 6.8 |
| 2016-Bter-CLO-C27-4-W1-head.D703\_D503.54244286.s\_fc.R1 |  |  |  |  |  |  |  |  |  |  | 37.0% | 40% | 76 | 17% | 1.5 |
| 2016-Bter-CLO-C27-4-W1-head.D703\_D503.HM3WHBBXX.s\_6.R1 |  |  |  |  |  |  |  |  |  |  | 60.6% | 41% | 50 | 33% | 2.5 |
| 2016-Bter-CLO-C27-4-W1-head.D703\_D503.HM3Y5BBXX.s\_6.R1 |  |  |  |  |  |  |  |  |  |  | 54.8% | 41% | 50 | 25% | 2.9 |
| 2016-Bter-CLO-C27-4-W1-head.D703\_D503.HMWT2BBXX.s\_3.R1 |  |  |  |  |  |  |  |  |  |  | 58.5% | 41% | 50 | 33% | 3.0 |
| 2016-Bter-CLO-C38-4-Q-head.D707\_D504.54237325.s\_fc.R1 |  |  |  |  |  |  |  |  |  |  | 58.7% | 43% | 76 | 25% | 2.9 |
| 2016-Bter-CLO-C38-4-Q-head.D707\_D504.HM3WHBBXX.s\_6.R1 |  |  |  |  |  |  |  |  |  |  | 78.9% | 44% | 50 | 33% | 5.0 |
| 2016-Bter-CLO-C38-4-Q-head.D707\_D504.HM3Y5BBXX.s\_6.R1 |  |  |  |  |  |  |  |  |  |  | 76.4% | 44% | 50 | 25% | 6.8 |
| 2016-Bter-CLO-C38-4-Q-head.D707\_D504.HMWT2BBXX.s\_3.R1 |  |  |  |  |  |  |  |  |  |  | 78.7% | 44% | 50 | 33% | 7.4 |
| 2016-Bter-CLO-C38-4-W1-head.D705\_D505.54249195.s\_fc.R1 |  |  |  |  |  |  |  |  |  |  | 46.6% | 41% | 76 | 17% | 1.9 |
| 2016-Bter-CLO-C38-4-W1-head.D705\_D505.HM3WHBBXX.s\_6.R1 |  |  |  |  |  |  |  |  |  |  | 68.5% | 42% | 50 | 33% | 3.2 |
| 2016-Bter-CLO-C38-4-W1-head.D705\_D505.HM3Y5BBXX.s\_6.R1 |  |  |  |  |  |  |  |  |  |  | 69.1% | 43% | 50 | 25% | 6.4 |
| 2016-Bter-CLO-C38-4-W1-head.D705\_D505.HMWT2BBXX.s\_3.R1 |  |  |  |  |  |  |  |  |  |  | 71.8% | 43% | 50 | 33% | 6.8 |
| 2016-Bter-CLO-C67-4-Q-head.D710\_D501.54248200.s\_fc.R1 |  |  |  |  |  |  |  |  |  |  | 64.0% | 41% | 76 | 25% | 12.3 |
| 2016-Bter-CLO-C67-4-Q-head.D710\_D501.HM3WHBBXX.s\_6.R1 |  |  |  |  |  |  |  |  |  |  | 63.2% | 42% | 50 | 33% | 1.9 |
| 2016-Bter-CLO-C67-4-Q-head.D710\_D501.HM3Y5BBXX.s\_6.R1 |  |  |  |  |  |  |  |  |  |  | 59.5% | 42% | 50 | 25% | 2.5 |
| 2016-Bter-CLO-C67-4-Q-head.D710\_D501.HMWT2BBXX.s\_3.R1 |  |  |  |  |  |  |  |  |  |  | 63.8% | 42% | 50 | 33% | 2.6 |
| 2016-Bter-CLO-C67-4-W1-head.D710\_D505.54243308.s\_fc.R1 |  |  |  |  |  |  |  |  |  |  | 61.6% | 42% | 76 | 25% | 6.8 |
| 2016-Bter-CLO-C67-4-W1-head.D710\_D505.HM3WHBBXX.s\_6.R1 |  |  |  |  |  |  |  |  |  |  | 75.3% | 43% | 50 | 33% | 6.3 |
| 2016-Bter-CLO-C67-4-W1-head.D710\_D505.HM3Y5BBXX.s\_6.R1 |  |  |  |  |  |  |  |  |  |  | 62.3% | 43% | 50 | 25% | 2.0 |
| 2016-Bter-CLO-C67-4-W1-head.D710\_D505.HMWT2BBXX.s\_3.R1 |  |  |  |  |  |  |  |  |  |  | 66.6% | 43% | 50 | 33% | 2.1 |
| 2016-Bter-CON-C06-4-Q-head.D710\_D506.54250196.s\_fc.R1 |  |  |  |  |  |  |  |  |  |  | 53.8% | 41% | 76 | 25% | 3.9 |
| 2016-Bter-CON-C06-4-Q-head.D710\_D506.HM3WHBBXX.s\_6.R1 |  |  |  |  |  |  |  |  |  |  | 65.7% | 42% | 50 | 25% | 2.1 |
| 2016-Bter-CON-C06-4-Q-head.D710\_D506.HM3Y5BBXX.s\_6.R1 |  |  |  |  |  |  |  |  |  |  | 69.6% | 42% | 50 | 25% | 7.1 |
| 2016-Bter-CON-C06-4-Q-head.D710\_D506.HMWT2BBXX.s\_3.R1 |  |  |  |  |  |  |  |  |  |  | 72.0% | 42% | 50 | 33% | 7.3 |
| 2016-Bter-CON-C06-4-W1-head.D708\_D505.54232438.s\_fc.R1 |  |  |  |  |  |  |  |  |  |  | 54.3% | 42% | 76 | 25% | 4.4 |
| 2016-Bter-CON-C06-4-W1-head.D708\_D505.HM3WHBBXX.s\_6.R1 |  |  |  |  |  |  |  |  |  |  | 74.7% | 43% | 50 | 33% | 8.6 |
| 2016-Bter-CON-C06-4-W1-head.D708\_D505.HM3Y5BBXX.s\_6.R1 |  |  |  |  |  |  |  |  |  |  | 59.8% | 43% | 50 | 25% | 2.4 |
| 2016-Bter-CON-C06-4-W1-head.D708\_D505.HMWT2BBXX.s\_3.R1 |  |  |  |  |  |  |  |  |  |  | 64.1% | 43% | 50 | 33% | 2.6 |
| 2016-Bter-CON-C34-4-Q-head.D707\_D507.54248195.s\_fc.R1 |  |  |  |  |  |  |  |  |  |  | 60.1% | 41% | 76 | 25% | 10.0 |
| 2016-Bter-CON-C34-4-Q-head.D707\_D507.HM3WHBBXX.s\_6.R1 |  |  |  |  |  |  |  |  |  |  | 71.3% | 42% | 50 | 33% | 7.5 |
| 2016-Bter-CON-C34-4-Q-head.D707\_D507.HM3Y5BBXX.s\_6.R1 |  |  |  |  |  |  |  |  |  |  | 59.0% | 42% | 50 | 25% | 2.1 |
| 2016-Bter-CON-C34-4-Q-head.D707\_D507.HMWT2BBXX.s\_3.R1 |  |  |  |  |  |  |  |  |  |  | 60.6% | 42% | 50 | 25% | 2.0 |
| 2016-Bter-CON-C34-4-W3-head.D704\_D501.54242294.s\_fc.R1 |  |  |  |  |  |  |  |  |  |  | 52.2% | 41% | 76 | 25% | 4.3 |
| 2016-Bter-CON-C34-4-W3-head.D704\_D501.HM3WHBBXX.s\_6.R1 |  |  |  |  |  |  |  |  |  |  | 66.9% | 42% | 50 | 33% | 4.8 |
| 2016-Bter-CON-C34-4-W3-head.D704\_D501.HM3Y5BBXX.s\_6.R1 |  |  |  |  |  |  |  |  |  |  | 59.5% | 42% | 50 | 25% | 2.8 |
| 2016-Bter-CON-C34-4-W3-head.D704\_D501.HMWT2BBXX.s\_3.R1 |  |  |  |  |  |  |  |  |  |  | 61.5% | 42% | 50 | 33% | 2.7 |
| 2016-Bter-CON-C48-4-Q-head.D703\_D505.54238313.s\_fc.R1 |  |  |  |  |  |  |  |  |  |  | 43.6% | 40% | 76 | 17% | 1.7 |
| 2016-Bter-CON-C48-4-Q-head.D703\_D505.HM3WHBBXX.s\_6.R1 |  |  |  |  |  |  |  |  |  |  | 58.1% | 41% | 50 | 25% | 0.8 |
| 2016-Bter-CON-C48-4-Q-head.D703\_D505.HM3Y5BBXX.s\_6.R1 |  |  |  |  |  |  |  |  |  |  | 69.2% | 41% | 50 | 25% | 8.4 |
| 2016-Bter-CON-C48-4-Q-head.D703\_D505.HMWT2BBXX.s\_3.R1 |  |  |  |  |  |  |  |  |  |  | 71.2% | 41% | 50 | 33% | 8.6 |
| 2016-Bter-CON-C48-4-W2-head.D712\_D501.54241300.s\_fc.R1 |  |  |  |  |  |  |  |  |  |  | 58.6% | 41% | 76 | 25% | 7.1 |
| 2016-Bter-CON-C48-4-W2-head.D712\_D501.HM3WHBBXX.s\_6.R1 |  |  |  |  |  |  |  |  |  |  | 69.8% | 42% | 50 | 33% | 5.1 |
| 2016-Bter-CON-C48-4-W2-head.D712\_D501.HM3Y5BBXX.s\_6.R1 |  |  |  |  |  |  |  |  |  |  | 59.1% | 42% | 50 | 25% | 2.2 |
| 2016-Bter-CON-C48-4-W2-head.D712\_D501.HMWT2BBXX.s\_3.R1 |  |  |  |  |  |  |  |  |  |  | 61.9% | 42% | 50 | 25% | 2.1 |
| 2016-Bter-CON-C61-4-Q-head.D705\_D501.54234359.s\_fc.R1 |  |  |  |  |  |  |  |  |  |  | 57.4% | 41% | 76 | 25% | 5.9 |
| 2016-Bter-CON-C61-4-Q-head.D705\_D501.HM3WHBBXX.s\_6.R1 |  |  |  |  |  |  |  |  |  |  | 73.6% | 42% | 50 | 33% | 8.1 |
| 2016-Bter-CON-C61-4-Q-head.D705\_D501.HM3Y5BBXX.s\_6.R1 |  |  |  |  |  |  |  |  |  |  | 66.2% | 42% | 50 | 25% | 4.4 |
| 2016-Bter-CON-C61-4-Q-head.D705\_D501.HMWT2BBXX.s\_3.R1 |  |  |  |  |  |  |  |  |  |  | 67.9% | 42% | 50 | 33% | 4.2 |
| 2016-Bter-CON-C61-4-W1-head.D706\_D505.54247263.s\_fc.R1 |  |  |  |  |  |  |  |  |  |  | 50.6% | 41% | 76 | 25% | 3.4 |
| 2016-Bter-CON-C61-4-W1-head.D706\_D505.HM3WHBBXX.s\_6.R1 |  |  |  |  |  |  |  |  |  |  | 63.7% | 42% | 50 | 33% | 1.9 |
| 2016-Bter-CON-C61-4-W1-head.D706\_D505.HM3Y5BBXX.s\_6.R1 |  |  |  |  |  |  |  |  |  |  | 66.3% | 42% | 50 | 25% | 5.2 |
| 2016-Bter-CON-C61-4-W1-head.D706\_D505.HMWT2BBXX.s\_3.R1 |  |  |  |  |  |  |  |  |  |  | 68.7% | 42% | 50 | 33% | 5.3 |
| 2016-Bter-IMI-C07-4-Q-head.D712\_D505.54239332.s\_fc.R1 |  |  |  |  |  |  |  |  |  |  | 53.9% | 42% | 76 | 25% | 3.9 |
| 2016-Bter-IMI-C07-4-Q-head.D712\_D505.HM3WHBBXX.s\_6.R1 |  |  |  |  |  |  |  |  |  |  | 68.4% | 43% | 50 | 33% | 3.6 |
| 2016-Bter-IMI-C07-4-Q-head.D712\_D505.HM3Y5BBXX.s\_6.R1 |  |  |  |  |  |  |  |  |  |  | 69.3% | 43% | 50 | 25% | 6.6 |
| 2016-Bter-IMI-C07-4-Q-head.D712\_D505.HMWT2BBXX.s\_3.R1 |  |  |  |  |  |  |  |  |  |  | 70.8% | 43% | 50 | 33% | 6.5 |
| 2016-Bter-IMI-C07-4-W4-head.D706\_D502.54248196.s\_fc.R1 |  |  |  |  |  |  |  |  |  |  | 56.7% | 41% | 76 | 25% | 6.2 |
| 2016-Bter-IMI-C07-4-W4-head.D706\_D502.HM3WHBBXX.s\_6.R1 |  |  |  |  |  |  |  |  |  |  | 64.8% | 42% | 50 | 33% | 2.6 |
| 2016-Bter-IMI-C07-4-W4-head.D706\_D502.HM3Y5BBXX.s\_6.R1 |  |  |  |  |  |  |  |  |  |  | 67.7% | 42% | 50 | 25% | 6.0 |
| 2016-Bter-IMI-C07-4-W4-head.D706\_D502.HMWT2BBXX.s\_3.R1 |  |  |  |  |  |  |  |  |  |  | 69.0% | 43% | 50 | 33% | 5.8 |
| 2016-Bter-IMI-C32-4-Q-head.D706\_D507.54238316.s\_fc.R1 |  |  |  |  |  |  |  |  |  |  | 53.6% | 41% | 76 | 25% | 4.0 |
| 2016-Bter-IMI-C32-4-Q-head.D706\_D507.HM3WHBBXX.s\_6.R1 |  |  |  |  |  |  |  |  |  |  | 65.9% | 42% | 50 | 33% | 2.9 |
| 2016-Bter-IMI-C32-4-Q-head.D706\_D507.HM3Y5BBXX.s\_6.R1 |  |  |  |  |  |  |  |  |  |  | 67.7% | 42% | 50 | 25% | 5.8 |
| 2016-Bter-IMI-C32-4-Q-head.D706\_D507.HMWT2BBXX.s\_3.R1 |  |  |  |  |  |  |  |  |  |  | 68.5% | 42% | 50 | 33% | 5.3 |
| 2016-Bter-IMI-C32-4-W1-head.D703\_D507.54247265.s\_fc.R1 |  |  |  |  |  |  |  |  |  |  | 56.8% | 40% | 76 | 25% | 5.9 |
| 2016-Bter-IMI-C32-4-W1-head.D703\_D507.HM3WHBBXX.s\_6.R1 |  |  |  |  |  |  |  |  |  |  | 62.0% | 41% | 50 | 25% | 1.7 |
| 2016-Bter-IMI-C32-4-W1-head.D703\_D507.HM3Y5BBXX.s\_6.R1 |  |  |  |  |  |  |  |  |  |  | 64.6% | 41% | 50 | 25% | 4.5 |
| 2016-Bter-IMI-C32-4-W1-head.D703\_D507.HMWT2BBXX.s\_3.R1 |  |  |  |  |  |  |  |  |  |  | 67.0% | 41% | 50 | 33% | 4.5 |
| 2016-Bter-IMI-C45-4-Q-head.D701\_D503.54244288.s\_fc.R1 |  |  |  |  |  |  |  |  |  |  | 48.8% | 41% | 76 | 17% | 2.4 |
| 2016-Bter-IMI-C45-4-Q-head.D701\_D503.HM3WHBBXX.s\_6.R1 |  |  |  |  |  |  |  |  |  |  | 70.3% | 42% | 50 | 33% | 4.9 |
| 2016-Bter-IMI-C45-4-Q-head.D701\_D503.HM3Y5BBXX.s\_6.R1 |  |  |  |  |  |  |  |  |  |  | 70.0% | 42% | 50 | 25% | 8.1 |
| 2016-Bter-IMI-C45-4-Q-head.D701\_D503.HMWT2BBXX.s\_3.R1 |  |  |  |  |  |  |  |  |  |  | 72.2% | 42% | 50 | 33% | 8.4 |
| 2016-Bter-IMI-C45-4-W1-head.D710\_D504.54244292.s\_fc.R1 |  |  |  |  |  |  |  |  |  |  | 55.3% | 41% | 76 | 25% | 7.2 |
| 2016-Bter-IMI-C45-4-W1-head.D710\_D504.HM3WHBBXX.s\_6.R1 |  |  |  |  |  |  |  |  |  |  | 63.4% | 42% | 50 | 33% | 3.5 |
| 2016-Bter-IMI-C45-4-W1-head.D710\_D504.HM3Y5BBXX.s\_6.R1 |  |  |  |  |  |  |  |  |  |  | 55.8% | 42% | 50 | 25% | 2.7 |
| 2016-Bter-IMI-C45-4-W1-head.D710\_D504.HMWT2BBXX.s\_3.R1 |  |  |  |  |  |  |  |  |  |  | 59.2% | 42% | 50 | 33% | 2.8 |
| 2016-Bter-IMI-C55-4-Q-head.D711\_D501.54239333.s\_fc.R1 |  |  |  |  |  |  |  |  |  |  | 64.5% | 41% | 76 | 25% | 11.7 |
| 2016-Bter-IMI-C55-4-Q-head.D711\_D501.HM3WHBBXX.s\_6.R1 |  |  |  |  |  |  |  |  |  |  | 67.5% | 42% | 50 | 33% | 3.0 |
| 2016-Bter-IMI-C55-4-Q-head.D711\_D501.HM3Y5BBXX.s\_6.R1 |  |  |  |  |  |  |  |  |  |  | 63.2% | 42% | 50 | 25% | 2.9 |
| 2016-Bter-IMI-C55-4-Q-head.D711\_D501.HMWT2BBXX.s\_3.R1 |  |  |  |  |  |  |  |  |  |  | 65.2% | 42% | 50 | 33% | 2.8 |
| 2016-Bter-IMI-C55-4-W1-head.D709\_D501.54234364.s\_fc.R1 |  |  |  |  |  |  |  |  |  |  | 60.7% | 43% | 76 | 25% | 6.9 |
| 2016-Bter-IMI-C55-4-W1-head.D709\_D501.HM3WHBBXX.s\_6.R1 |  |  |  |  |  |  |  |  |  |  | 61.5% | 43% | 50 | 25% | 1.0 |
| 2016-Bter-IMI-C55-4-W1-head.D709\_D501.HM3Y5BBXX.s\_6.R1 |  |  |  |  |  |  |  |  |  |  | 66.0% | 43% | 50 | 25% | 4.4 |
| 2016-Bter-IMI-C55-4-W1-head.D709\_D501.HMWT2BBXX.s\_3.R1 |  |  |  |  |  |  |  |  |  |  | 70.6% | 43% | 50 | 33% | 5.1 |
| CLO-C02.sorted\_queen |  |  |  |  |  |  |  | 96.3% | 16.9 | 17.6 |  |  |  |  |  |
| CLO-C02.sorted\_queen\_stats | 42% | 0.8% | 1.5% | 4.4% | 7.1% | 15.1% | 0.0 |  |  |  |  |  |  |  |  |
| CLO-C02.sorted\_worker |  |  |  |  |  |  |  | 96.6% | 19.2 | 19.9 |  |  |  |  |  |
| CLO-C02.sorted\_worker\_stats | 42% | 1.3% | 2.3% | 5.7% | 8.3% | 16.5% | 0.0 |  |  |  |  |  |  |  |  |
| CLO-C27.sorted\_queen |  |  |  |  |  |  |  | 96.8% | 18.6 | 19.2 |  |  |  |  |  |
| CLO-C27.sorted\_queen\_stats | 42% | 0.8% | 1.5% | 4.5% | 7.1% | 14.6% | 0.0 |  |  |  |  |  |  |  |  |
| CLO-C27.sorted\_worker |  |  |  |  |  |  |  | 96.3% | 9.7 | 10.1 |  |  |  |  |  |
| CLO-C27.sorted\_worker\_stats | 42% | 0.5% | 0.9% | 3.2% | 5.5% | 12.8% | 0.0 |  |  |  |  |  |  |  |  |
| CLO-C38.sorted\_queen |  |  |  |  |  |  |  | 97.4% | 22.7 | 23.3 |  |  |  |  |  |
| CLO-C38.sorted\_queen\_stats | 45% | 1.1% | 1.8% | 4.2% | 6.2% | 12.3% | 0.0 |  |  |  |  |  |  |  |  |
| CLO-C38.sorted\_worker |  |  |  |  |  |  |  | 97.5% | 18.3 | 18.8 |  |  |  |  |  |
| CLO-C38.sorted\_worker\_stats | 43% | 0.9% | 1.6% | 4.3% | 6.6% | 13.2% | 0.0 |  |  |  |  |  |  |  |  |
| CLO-C67.sorted\_queen |  |  |  |  |  |  |  | 96.0% | 18.9 | 19.7 |  |  |  |  |  |
| CLO-C67.sorted\_queen\_stats | 42% | 1.3% | 2.3% | 5.9% | 8.6% | 17.0% | 0.0 |  |  |  |  |  |  |  |  |
| CLO-C67.sorted\_worker |  |  |  |  |  |  |  | 96.7% | 17.1 | 17.7 |  |  |  |  |  |
| CLO-C67.sorted\_worker\_stats | 43% | 1.0% | 1.8% | 4.5% | 6.7% | 13.3% | 0.0 |  |  |  |  |  |  |  |  |
| CON-C06.sorted\_queen |  |  |  |  |  |  |  | 97.2% | 20.5 | 21.1 |  |  |  |  |  |
| CON-C06.sorted\_queen\_stats | 43% | 1.0% | 1.9% | 5.2% | 7.9% | 15.5% | 0.0 |  |  |  |  |  |  |  |  |
| CON-C06.sorted\_worker |  |  |  |  |  |  |  | 96.8% | 18.2 | 18.8 |  |  |  |  |  |
| CON-C06.sorted\_worker\_stats | 43% | 1.1% | 1.9% | 4.7% | 7.0% | 13.7% | 0.0 |  |  |  |  |  |  |  |  |
| CON-C34.sorted\_queen |  |  |  |  |  |  |  | 96.6% | 21.5 | 22.2 |  |  |  |  |  |
| CON-C34.sorted\_queen\_stats | 43% | 1.4% | 2.5% | 6.3% | 9.0% | 17.4% | 0.0 |  |  |  |  |  |  |  |  |
| CON-C34.sorted\_worker |  |  |  |  |  |  |  | 96.5% | 14.5 | 15.0 |  |  |  |  |  |
| CON-C34.sorted\_worker\_stats | 42% | 0.8% | 1.5% | 4.2% | 6.6% | 13.9% | 0.0 |  |  |  |  |  |  |  |  |
| CON-C48.sorted\_queen |  |  |  |  |  |  |  | 97.0% | 19.7 | 20.3 |  |  |  |  |  |
| CON-C48.sorted\_queen\_stats | 42% | 1.0% | 1.7% | 4.9% | 7.6% | 15.0% | 0.0 |  |  |  |  |  |  |  |  |
| CON-C48.sorted\_worker |  |  |  |  |  |  |  | 96.2% | 16.5 | 17.1 |  |  |  |  |  |
| CON-C48.sorted\_worker\_stats | 43% | 1.0% | 1.8% | 4.8% | 7.2% | 14.4% | 0.0 |  |  |  |  |  |  |  |  |
| CON-C61.sorted\_queen |  |  |  |  |  |  |  | 95.9% | 22.4 | 23.4 |  |  |  |  |  |
| CON-C61.sorted\_queen\_stats | 42% | 1.2% | 2.2% | 5.7% | 8.6% | 16.8% | 0.0 |  |  |  |  |  |  |  |  |
| CON-C61.sorted\_worker |  |  |  |  |  |  |  | 96.9% | 15.9 | 16.4 |  |  |  |  |  |
| CON-C61.sorted\_worker\_stats | 42% | 0.8% | 1.5% | 4.3% | 6.7% | 13.7% | 0.0 |  |  |  |  |  |  |  |  |
| IMI-C07.sorted\_queen |  |  |  |  |  |  |  | 97.0% | 20.6 | 21.2 |  |  |  |  |  |
| IMI-C07.sorted\_queen\_stats | 44% | 1.0% | 1.9% | 5.0% | 7.6% | 15.1% | 0.0 |  |  |  |  |  |  |  |  |
| IMI-C07.sorted\_worker |  |  |  |  |  |  |  | 96.8% | 20.5 | 21.2 |  |  |  |  |  |
| IMI-C07.sorted\_worker\_stats | 43% | 1.1% | 2.0% | 5.3% | 7.8% | 15.7% | 0.0 |  |  |  |  |  |  |  |  |
| IMI-C32.sorted\_queen |  |  |  |  |  |  |  | 96.9% | 18.1 | 18.6 |  |  |  |  |  |
| IMI-C32.sorted\_queen\_stats | 43% | 0.9% | 1.5% | 4.6% | 7.4% | 15.4% | 0.0 |  |  |  |  |  |  |  |  |
| IMI-C32.sorted\_worker |  |  |  |  |  |  |  | 96.5% | 16.5 | 17.1 |  |  |  |  |  |
| IMI-C32.sorted\_worker\_stats | 42% | 1.0% | 1.7% | 4.5% | 6.8% | 13.9% | 0.0 |  |  |  |  |  |  |  |  |
| IMI-C45.sorted\_queen |  |  |  |  |  |  |  | 97.5% | 24.1 | 24.7 |  |  |  |  |  |
| IMI-C45.sorted\_queen\_stats | 43% | 1.2% | 2.1% | 5.3% | 7.9% | 15.0% | 0.0 |  |  |  |  |  |  |  |  |
| IMI-C45.sorted\_worker |  |  |  |  |  |  |  | 96.3% | 16.0 | 16.6 |  |  |  |  |  |
| IMI-C45.sorted\_worker\_stats | 42% | 1.1% | 2.1% | 5.2% | 7.6% | 14.9% | 0.0 |  |  |  |  |  |  |  |  |
| IMI-C55.sorted\_queen |  |  |  |  |  |  |  | 94.9% | 20.1 | 21.2 |  |  |  |  |  |
| IMI-C55.sorted\_queen\_stats | 42% | 1.2% | 2.2% | 6.0% | 8.9% | 17.5% | 0.0 |  |  |  |  |  |  |  |  |
| IMI-C55.sorted\_worker |  |  |  |  |  |  |  | 96.9% | 17.3 | 17.8 |  |  |  |  |  |
| IMI-C55.sorted\_worker\_stats | 44% | 1.1% | 1.9% | 4.6% | 6.9% | 14.1% | 0.0 |  |  |  |  |  |  |  |  |

×

#### General Statistics: Columns

Uncheck the tick box to hide columns. Click and drag the handle on the left to change order.

Show All
Show None

| Sort | Visible | Group | Column | Description | ID | Scale |
| --- | --- | --- | --- | --- | --- | --- |
| || |  | QualiMap | Avg. GC | Average GC content | `avg_gc` |  |
| || |  | QualiMap | ≥ 50X | Fraction of genome with at least 50X coverage | `fifty_x_pc` |  |
| || |  | QualiMap | ≥ 30X | Fraction of genome with at least 30X coverage | `thirty_x_pc` |  |
| || |  | QualiMap | ≥ 10X | Fraction of genome with at least 10X coverage | `ten_x_pc` |  |
| || |  | QualiMap | ≥ 05X | Fraction of genome with at least 05X coverage | `five_x_pc` |  |
| || |  | QualiMap | ≥ 01X | Fraction of genome with at least 01X coverage | `one_x_pc` |  |
| || |  | QualiMap | Coverage | Median coverage | `median_coverage` |  |
| || |  | QualiMap | % Aligned | % mapped reads | `percentage_aligned` |  |
| || |  | QualiMap | Aligned | Number of mapped reads (millions) | `mapped_reads` | read\_count |
| || |  | QualiMap | Total Reads | Number of reads (millions) | `total_reads` | read\_count |
| || |  | FastQC | % Dups | % Duplicate Reads | `percent_duplicates` |  |
| || |  | FastQC | % GC | Average % GC Content | `percent_gc` |  |
| || |  | FastQC | Length | Average Sequence Length (bp) | `avg_sequence_length` |  |
| || |  | FastQC | % Failed | Percentage of modules failed in FastQC report (includes those not plotted here) | `percent_fails` |  |
| || |  | FastQC | M Seqs | Total Sequences (millions) | `total_sequences` | read\_count |

Close

## QualiMap

QualiMap is a platform-independent application to facilitate the quality control of alignment sequencing data and its derivatives like feature counts.

### Coverage Histogram

loading..

---

### Genome Fraction Coverage

loading..

---

### GC-content distribution

loading..

---

## FastQC

FastQC is a quality control tool for high throughput sequence data, written by Simon Andrews at the Babraham Institute in Cambridge.

### Sequence Quality Histograms

The mean quality value across each base position in the read. See the FastQC help.

loading..

---

### Per Sequence Quality Scores

The number of reads with average quality scores. Shows if a subset of reads has poor quality. See the FastQC help.

loading..

---

### Per Base Sequence Content

The proportion of each base position for which each of the four normal DNA bases has been called. See the FastQC help.

Click a heatmap row to see a line plot for that dataset.

##### *rollover for sample name*

Position: -

%T: -

%C: -

%A: -

%G: -

---

### Per Sequence GC Content

The average GC content of reads. Normal random library typically have a roughly normal distribution of GC content. See the FastQC help.

Percentages
Counts

loading..

---

### Per Base N Content

The percentage of base calls at each position for which an N was called. See the FastQC help.

loading..

---

### Sequence Length Distribution

The distribution of fragment sizes (read lengths) found. See the FastQC help.

loading..

---

### Sequence Duplication Levels

The relative level of duplication found for every sequence. See the FastQC help.

loading..

---

### Adapter Content

The cumulative percentage count of the proportion of your library which has seen each of the adapter sequences at each position. See the FastQC help. Only samples with ≥ 0.1% adapter contamination are shown.

loading..

**MultiQC v0.7**
- Written by Phil Ewels,
available on GitHub.

This report uses HighCharts,
jQuery,
jQuery UI,
Bootstrap,
chroma.js,
FileSaver.js and
clipboard.js.

×

### Regex Help

Toolbox search strings can behave as regular expressions (regexes). Click a button below to see an example of it in action. Try modifying them yourself in the text box.

`^` (start of string)
`$` (end of string)
`[]` (character choice)
`\d` (shorthand for `[0-9]`)
`\w` (shorthand for `[0-9a-zA-Z_]`)
`.` (any character)
`\.` (literal full stop)
`()` `|` (group / separator)
`*` (prev char 0 or more)
`+` (prev char 1 or more)
`?` (prev char 0 or 1)
`{}` (char num times)
`{,}` (count range)

```
samp_1
samp_1_edited
samp_2
samp_2_edited
samp_3
samp_3_edited
prepended_samp_1
tmp_samp_1_edited
tmpp_samp_1_edited
tmppp_samp_1_edited
#samp_1_edited.tmp
samp_11
samp_11111
```

See regex101.com for a more heavy duty testing suite.

Close
