# Supplementary material for: Caste‐ and pesticide‐specific effects of neonicotinoid pesticide exposure on gene expression in bumblebees
Source: Mol Ecol. 2019 Mar 6;28(8):1964–74. doi: 10.1111/mec.15047 (PMC6563198; doi:10.1111/mec.15047)
Supplement: Supplementary file 8 [file MEC-28-1964-s008.docx]

**Supplemental Information for:**

Caste- and pesticide-specific effects of neonicotinoid pesticide exposure on gene expression in bumblebees

Thomas J. Colgan, Isabel K. Fletcher, Andres N. Arce, Richard J. Gill, Ana Ramos Rodrigues, Eckart Stolle, Lars Chittka and Yannick Wurm

**Table of Contents:**

| **Comparing kallisto pseudoalignment and HISAT2 mapping-based quantification of reads to detect differential expression** | Page 2-3 |
| --- | --- |
| **Supplemental Figure S1** | Page 4 |
| **Supplemental Figure S2** | Page 5 |
| **Supplemental Figure S3** | Page 6 |
| **Supplemental Figure S4** | Page 7 |
| **Supplemental Figure S5** | Page 8 |

## Comparing kallisto pseudoalignment and HISAT2 mapping-based quantification of reads to detect differential expression

We investigated whether exposure to two common neonicotinoid pesticides affects gene expression amplitude or alternative splicing in heads of bumblebee workers and queens. To identify differentially expressed transcripts, we first estimated read counts using the *k*-mer based probabilistic pseudoaligner kallisto. Kallisto has been cited ~1,000 times since publication in 2016, including to detect differential expression in many high profile biomedical studies (e.g., [(Edfors *et al.* 2016; Mukherjee *et al.* 2017; Thul *et al.* 2017; Mumbach *et al.* 2017; Lachmann *et al.* 2018)](https://paperpile.com/c/P8VeBX/uLEZ+35cs+tPSw+GDcH+Z4Xr). Importantly, a comparative analysis of RNA-Seq quantification tools demonstrated that pseudoaligners such as kallisto have higher accuracy and consistency in transcript quantification than traditional aligners [(Sahraeian *et al.* 2017)](https://paperpile.com/c/P8VeBX/Stkc). To detect differential expression based on kallisto estimated counts, we used DESeq2, one of the most widely used packages for such analyses [(Love *et al.* 2014](https://paperpile.com/c/P8VeBX/7NmMK); cited >7,000 times). Unfortunately, DESeq2 cannot perform differential exon usage analysis. We thus asked the lead author of DESeq2, Dr. Michael Love what approach he recommends for differential exon usage (<https://support.bioconductor.org/p/108576/>). We followed his advice to use HISAT2/DEXSeq. Specifically, we used the splice-aware aligner HISAT2 [(Kim *et al.* 2015)](https://paperpile.com/c/P8VeBX/qnJs) to align our data against the reference bumblebee genome. From the resulting SAM alignment files, we extracted exon-level read counts using HTSeq [(Anders *et al.* 2015)](https://paperpile.com/c/P8VeBX/sdcE). We finally provided these counts to DEXSeq [(Reyes *et al.* 2013)](https://paperpile.com/c/P8VeBX/wO8k) to detect differential exon usage.

As a measure of confidence to determine whether our differential expression amplitude results are robust to analytical approach, we performed a redundant analysis using gene-level read counts extracted using HTSeq from HISAT2-aligned SAM files. Similarly to the kallisto-based analysis, we used these counts as input for DESeq2 to identify differentially expressed genes. We compared findings of the two approaches at three levels: general trends of relative magnitudes of gene expression, specific genes identified, and Gene Ontology categories. For all three levels, we found that the kallisto- and HISAT2-based approaches gave highly similar results. We provide details for each comparison below.

**Overall trends:** Similar to the kallisto-based analysis, the HISAT2-based approach identified caste- and pesticide-specific effects of treatments, with clothianidin having stronger effects on gene expression amplitudes than imidacloprid. Furthermore, in response to clothianidin exposure, we find stronger effects on workers (n = 60 differentially expressed genes) than on queens (n = 18), in line with our kallisto-based analysis. The HISAT2-based approach found no effects of imidacloprid exposure on gene expression in either caste.

**Specific Genes:** 88% of the 17 genes identified as differentially expressed in clothianidin-exposed queens using the kallisto approach were also significant in the HISAT2-based analysis (see Euler Diagram in Supp. Fig. S4(A)). Similarly, 80% of the 55 genes identified as differentially expressed in clothianidin-exposed workers using the kallisto-based approach were also significant in the HISAT2-based analysis (see Euler Diagram in Supp. Fig. S4(B)).

Some of the genes detected as differentially expressed using the kallisto-based approach are explicitly mentioned in the Results or Discussion text. Here, for each of these genes, we indicate significance levels in the HISAT2-based analysis.

In the Results:

- The eight genes mentioned as being differentially expressed only after clothianidin exposure were detected using both approaches.
- However, one additional gene (LOC100644101) which was differentially expressed in workers in response to both pesticides in the kallisto-based analysis was only identified as significantly differentially expressed in clothianidin-exposed workers by the HISAT2-based approach. The effect of imidacloprid on this gene was marginally non-significant (BH adjusted *p-*value = 0.072) using the HISAT2-based approach.

In the Discussion:

- Two of the genes mentioned by name (*alanine-glyoxylate aminotransferase*: LOC100650318 and *alanine-glyoxylate aminotransferase*: LOC100631088) were differentially expressed according to both approaches.
- Of the three putative cytochrome P450 genes discussed, two (LOC100652170; LOC100649441) were detected as differentially expressed by both approaches while one gene (LOC100648391) was identified as non-significant (BH adjusted *p*-value = 0.176) by the HISAT2-based approach.
- Among the six genes we discuss as being affected by other pesticides in other studies, four were significant in both analyses, while the remaining two genes were marginally non-significant in the HISAT2-based analysis (*calponin*, BH adjusted *p-*value = 0.06; *hexosaminidase D*, BH adjusted *p*-value = 0.058).

**Gene Ontology:** Lastly, we compared Gene Ontology (GO) enrichment analyses obtained from the two analysis approaches. Because the relatively conservative topGO algorithm ‘weight01’ removes Gene Ontology terms in an analysis-specific manner to increase sensitivity and specificity, we instead performed these comparisons with the topGO ‘classic’ algorithm, which reports all Gene Ontology terms. For clothianidin-exposed queens, 48 GO terms were significant according to both approaches, representing 74% of the 65 terms identified in the kallisto-based analysis and 96% of the 50 terms identified in the HISAT2-based analysis (see Euler Diagram in Supp. Fig. S5(A)). Similarly, for clothianidin-exposed workers, 18 GO terms were significant according to both approaches, representing 69.2% of the 24 terms identified in the kallisto-based analysis and 48.6% of the 37 terms identified in the HISAT2-based analysis (see Euler Diagram in Supp. Fig. S5(B)).

# Supplemental Figures

**Supp. Fig. S1. Experimental design for RNA-Seq pesticide exposure experiment.** Twelve entire bumblebee colonies of controlled size (see Materials and Methods) were randomly assigned to either a control, clothianidin or imidacloprid treatment. On day 1, between one and four newly eclosed bumblebee workers were briefly taken from the colony, tagged for future identification and returned to the colony. We provided all colonies for all treatments with sucrose solution *ad libitum*. Six days later, colonies assigned a neonicotinoid treatment were exposed to either clothianidin- or imidacloprid-laced sucrose solution while control colonies remained on untreated sucrose solution. After four days of exposure, we collected the 10 day old workers, as well as the queen for each colony. Collected individuals were immediately transferred to individual cryotubes and snap frozen in liquid nitrogen.


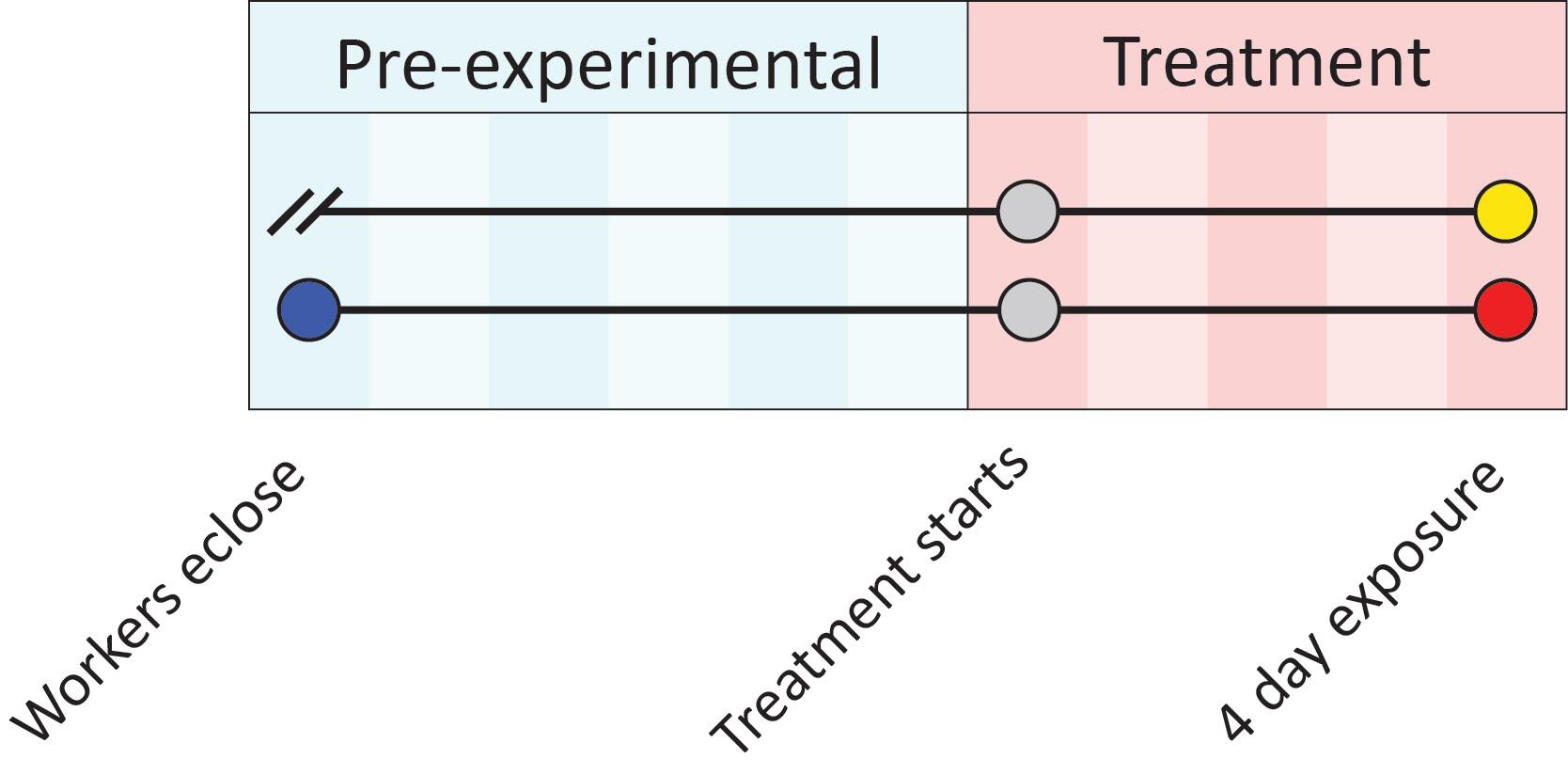


**Supp. Fig. S2. Gene expression changes in amplitude and splicing in response to neonicotinoid insecticides.** Euler diagram of the overlap among genes identified as significantly differentially expressed (DE) and/or alternatively spliced (splicing) in each caste (“Worker”; “Queen”) in response to one of two neonicotinoid treatments (“clothianidin” or “imidacloprid”).


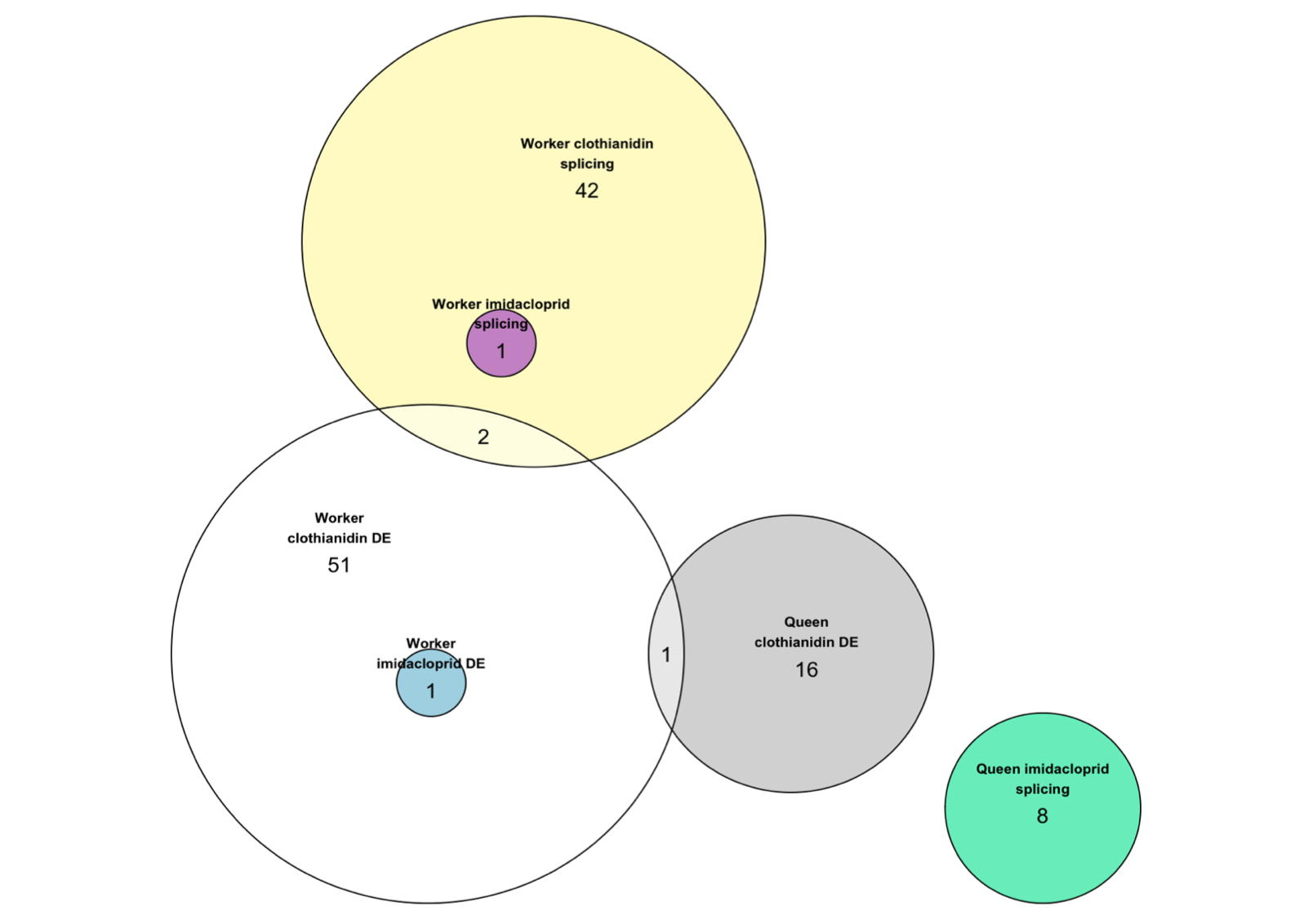


**Supp. Fig. S3. Enrichment of Gene Ontology terms associated with genes differentially expressed in response to clothianidin exposure.** Each bar represents the -log10 of *p*-value for a rank-based analysis (Kolmogorov-Smirnov test) for each GO term; in parentheses, we show the total number of annotated genes for each term. The vertical black line indicates a -log10(*p*) value equivalent to the *p*-value = 0.05 threshold of significance. Ontologies are colored in grey (molecular function), blue (cellular component) and orange (biological process).

**Supp. Fig. S4. High overlap of differentially expressed genes detected by DESeq2 using kallisto- and HISAT2-based approaches for each caste exposed to clothianidin.** Euler diagram of significant (BH adjusted *p*-value < 0.05) differentially expressed genes identified using two independent approaches: 1) “kallisto”: Genes identified as differentially expressed by DESeq2 when using estimated counts generated by the pseudoaligner kallisto as input; and 2) “HISAT2-HTSeq”: Genes identified as differentially expressed by DESeq2 when using actual gene-level counts generated by the HISAT2-HTSeq pipeline as input. Euler diagrams represent the overlap in significant differentially expressed genes detected by each approach for (A) clothianidin-exposed queens and (B) clothianidin-exposed workers.


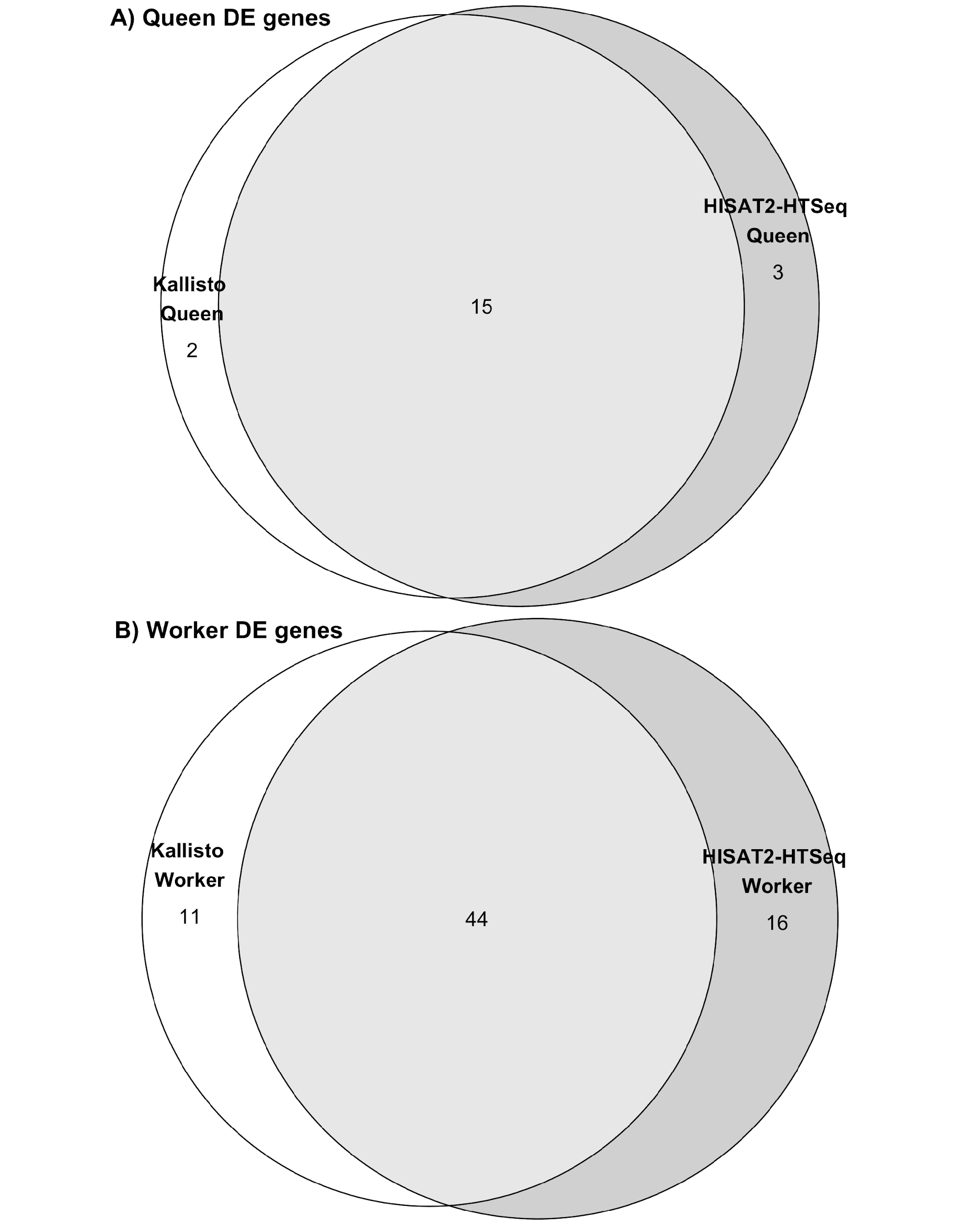


**Supp. Fig. S5. High overlap of enriched Gene Ontology terms detected by topGO using kallisto- and HISAT2-based approaches for each caste exposed to clothianidin.** Euler diagrams of significant (*p*-value < 0.05) enriched Gene Ontology terms identified by two independent approaches: 1) “kallisto”: Gene Ontology enrichment analysis performed by topGO on differentially expressed genes identified by DESeq2 when using estimated counts generated by the pseudoaligner kallisto as input; and 2) “HISAT2-HTSeq”: Gene Ontology enrichment analysis performed by topGO on differentially expressed genes identified by DESeq2 when using actual gene-level counts generated by the HISAT2-HTSeq pipeline as input. Euler diagrams represent the overlap in enriched Gene Ontology terms detected by each approach for (A) clothianidin-exposed queens and (B) clothianidin-exposed workers.


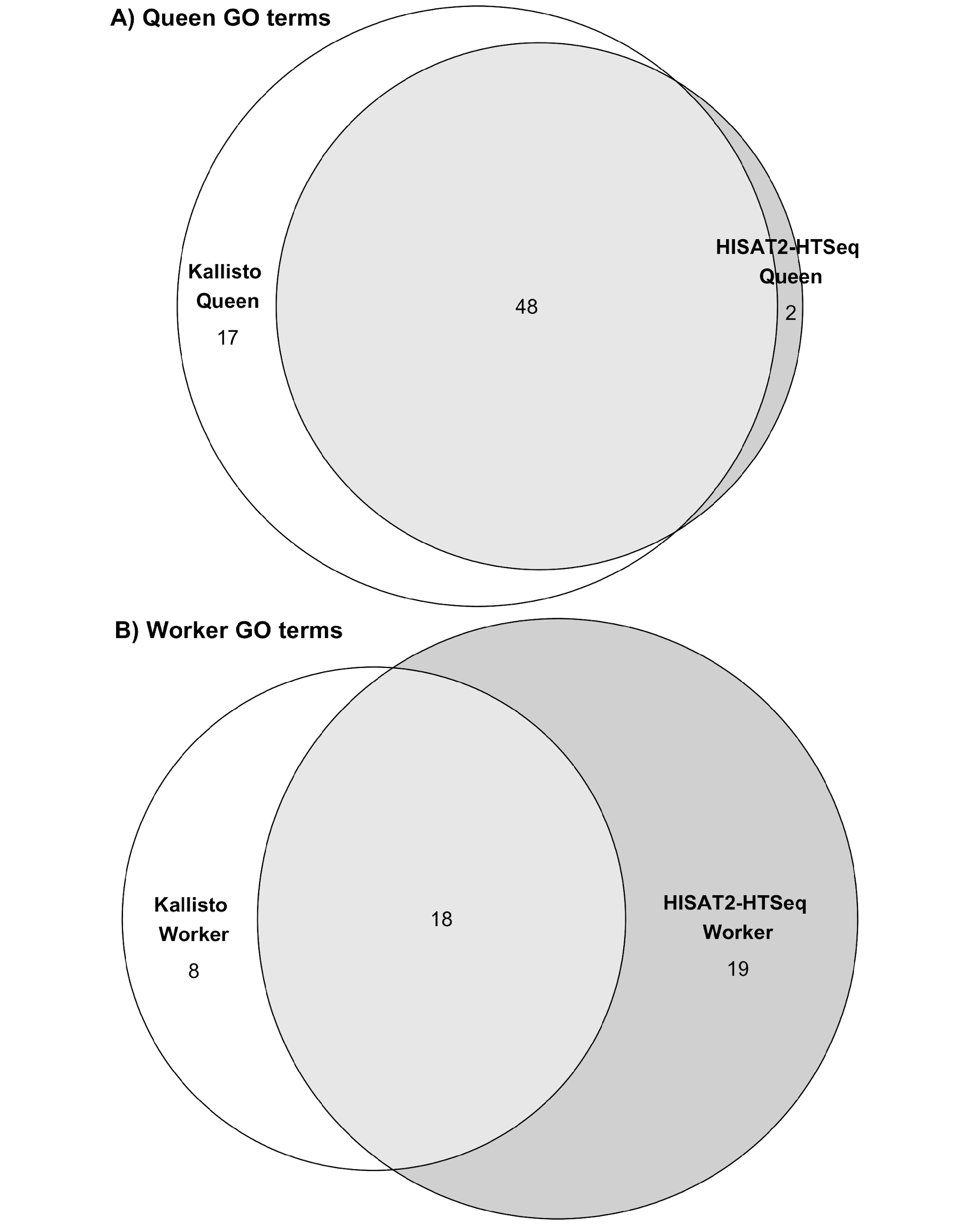


# References

[Anders S, Pyl PT, Huber W (2015) HTSeq--a Python framework to work with high-throughput sequencing data. *Bioinformatics* , **31**, 166–169.](http://paperpile.com/b/P8VeBX/sdcE)

[Edfors F, Danielsson F, Hallström BM *et al.* (2016) Gene‐specific correlation of RNA and protein levels in human cells and tissues. *Molecular Systems Biology*, **12**, 883.](http://paperpile.com/b/P8VeBX/GDcH)

[Kim D, Langmead B, Salzberg SL (2015) HISAT: a fast spliced aligner with low memory requirements. *Nature Methods*, **12**, 357–360.](http://paperpile.com/b/P8VeBX/qnJs)

[Lachmann A, Torre D, Keenan AB *et al.* (2018) Massive mining of publicly available RNA-seq data from human and mouse. *Nature Communications*, **9**, 1366.](http://paperpile.com/b/P8VeBX/35cs)

[Love MI, Huber W, Anders S (2014) Moderated estimation of fold change and dispersion for RNA-seq data with DESeq2. *Genome Biology*, **15**, 550.](http://paperpile.com/b/P8VeBX/7NmMK)

[Mukherjee N, Calviello L, Hirsekorn A *et al.* (2017) Integrative classification of human coding and noncoding genes through RNA metabolism profiles. *Nature Structural & Molecular Biology*, **24**, 86–96.](http://paperpile.com/b/P8VeBX/Z4Xr)

[Mumbach MR, Satpathy AT, Boyle EA *et al.* (2017) Enhancer connectome in primary human cells identifies target genes of disease-associated DNA elements. *Nature Genetics*, **49**, 1602–1612.](http://paperpile.com/b/P8VeBX/tPSw)

[Reyes A, Anders S, Weatheritt RJ *et al.* (2013) Drift and conservation of differential exon usage across tissues in primate species. *Proceedings of the National Academy of Sciences of the United States of America*, **110**, 15377–15382.](http://paperpile.com/b/P8VeBX/wO8k)

[Sahraeian SME, Mohiyuddin M, Sebra R *et al.* (2017) Gaining comprehensive biological insight into the transcriptome by performing a broad-spectrum RNA-seq analysis. *Nature Communications*, **8**, 59.](http://paperpile.com/b/P8VeBX/Stkc)

[Thul PJ, Åkesson L, Wiking M *et al.* (2017) A subcellular map of the human proteome. *Science*, **356**.](http://paperpile.com/b/P8VeBX/uLEZ)
